# “Central Road” cystoscopic finding: The road to worsened incontinence following laparoscopic sacrocolpopexy

**DOI:** 10.1002/iju5.12189

**Published:** 2020-07-01

**Authors:** Kumiko Kato, Yasukuni Yoshimura, Masahiro Narushima, Shoji Suzuki, Ryohei Hattori

**Affiliations:** ^1^ Department of Female Urology Japanese Red Cross Nagoya First Hospital Nagoya Japan; ^2^ Female Pelvic Health Center Showa University Northern Yokohama Hospital Yokohama Japan; ^3^ Department of Female Urology and Urogynecology Center Meitetsu Hospital Nagoya Japan; ^4^ Department of Urology Japanese Red Cross Nagoya First Hospital Nagoya Japan

**Keywords:** cystoscopy, cystourethrography, laparoscopic sacrocolpopexy, pelvic organ prolapse, stress urinary incontinence

## Abstract

**Introduction:**

This paper presents the “Central Road” cystoscopic finding accompanied by magnified mixed urinary incontinence following laparoscopic sacrocolopopexy.

**Case presentation:**

A 70‐year‐old female experienced severe mixed urinary incontinence upon completing laparoscopic sacrocolopopexy. The cystoscopy showed a cord‐like appearance in the center of the bladder trigon and posterior wall. Videourodynamics confirmed stress urinary incontinence, and chain cystourethrography indicated that the proximal urethra was open and the posterior vesicourethral angle was atypically widened. After implanting a midurethral sling, mixed urinary incontinence was cured subjectively and objectively without medication.

**Conclusion:**

The “Central Road” cystoscopic finding can be a signpost pointing to laparoscopic sacrocolopopexy mesh overtensioning, which can cause dekinking of the bladder neck, exacerbate stress urinary incontinence, and possibly lead to stress‐induced instability. A midurethral sling successfully relieved mixed urinary incontinence in this case, but it might be necessary to loosen the laparoscopic sacrocolopopexy mesh in some other cases.

**Abbreviations & Acronyms**
FDA: U.S. Food and Drug Administration
LSC: laparoscopic sacrocolpopexy
MUI: mixed urinary incontinence
POP‐Q: pelvic organ prolapse‐quantification
SUI: stress urinary incontinence
TVM: transvaginal mesh prolapse surgery
UUI: urgency urinary incontinence

**Keynote message**

In 2011, the FDA released an alert about mesh complications.^1^ Following the release, TVM decreased or was banned, then replaced by other prolapse surgeries including LSC in the United States and Europe. In Asian countries, although TVM remains as a leading surgical option due to relatively low complication rates, more doctors have introduced LSC, especially to younger or sexually active women.^2^ As the number of LSC is increasing, problems related with LSC are beginning to be uncovered. This paper presents a “Central Road” cystoscopic finding caused by overtensioning of the LSC mesh in a female who developed severe MUI after LSC.

## Case presentation

A 70‐year‐old woman underwent LSC due to POP‐Q stage III cystocele. Following subtotal hysterectomy, mesh was fixed to the anterior and posterior vaginal walls, and the uterine cervix was fixed to the sacrum with mesh. As the preoperative cough stress test during prolapse reduction was negative, concomitant anti‐incontinence surgery was not performed.

Although MUI only occurred occasionally before LSC, it severely worsened directly after LSC (SUI 5–6 times and UUI 1–2 times a day). She had urinary incontinence while sneezing, coughing, carrying her grandchildren, and during physical exercise such as brisk walking and jumping. With successive coughs, massive incontinence occurred with urinary urgency. It was necessary for her to change 80cc urinary pads four times a day.

**Fig. 1:**
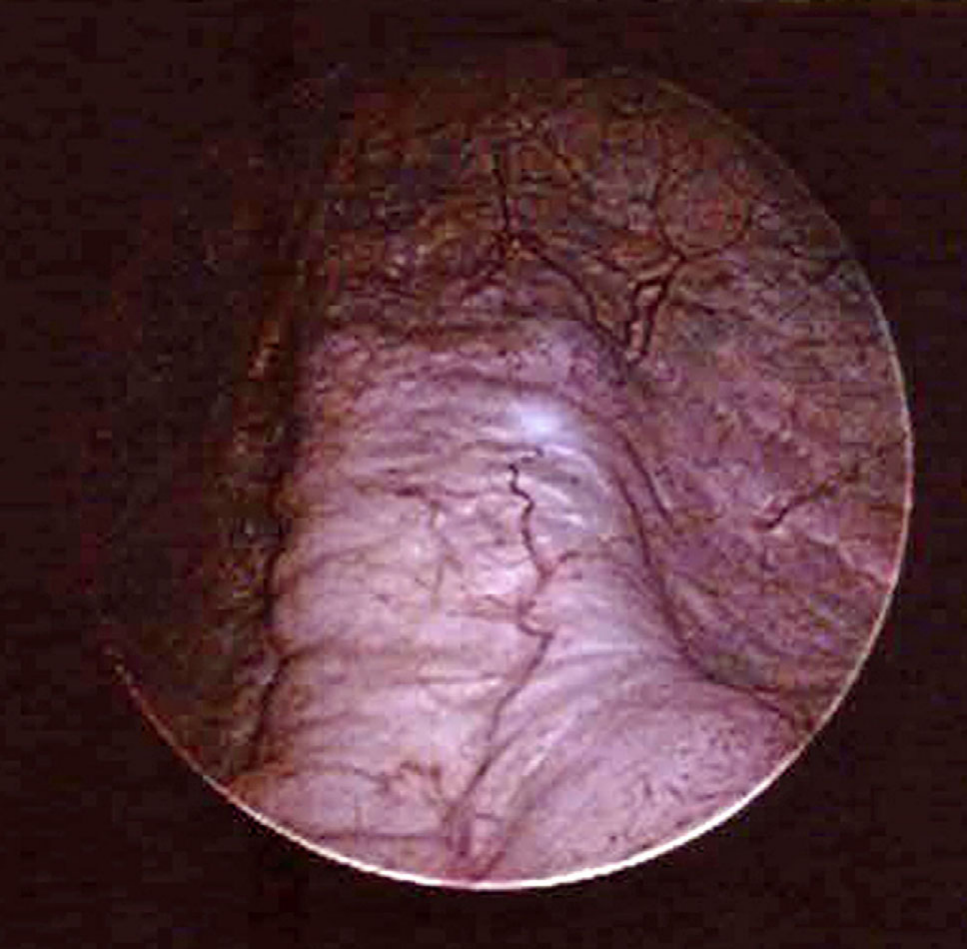
“Central Road” finding on a cystoscope, a cord‐like elevation in the center of the bladder trigon and posterior wall.

**Fig. 2:**
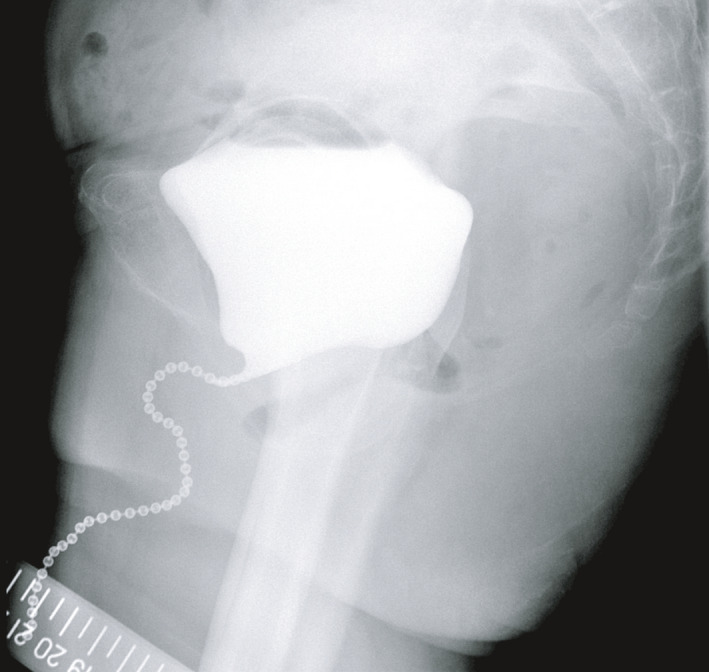
Lateral standing view of chain cystourethrogram. The bladder neck and proximal urethra were open, and both the upper urethral angle and the posterior vesicourethral angle were widened in an atypical way.

The patient and her family were informed beforehand of the risk of urinary incontinence worsening after prolapse surgery. However, they became quite distressed at the extent of the worsened incontinence. Anticholinergics decreased the amount of UUI but not SUI. During a cystoscopy, there was no mesh exposure or tumor, but an unusual cord‐like appearance was revealed in the center of the bladder trigon and posterior wall resembling a “Central Road” (Fig. 1). A stress test proved leakage accompanied by coughing and straining, and this leakage was prevented by supporting the paraurethral tissue with two fingers (Bonny test). A 1‐h pad test resulted in a leakage of 44 g/h. Uroflowmetry was normal (maximum flow rate 24 mL/s, voided volume 491 mL, and residual volume 22 mL). Videourodynamics verified SUI without detrusor overactivity under anticholinergic medication. Chain cystourethrography showed that the bladder neck was open, with the posterior vesicourethral angle widened atypically (Fig. 2).

Following LSC, the patient received a midurethral sling procedure (transobturator tape) 18 months later. Intraoperative cough stress tests were used to adjust the tape tension. During 20 months of follow‐ups, both SUI and UUI were completely cured without medication, and urinary pads were not needed. Cough stress tests and 1‐h pad tests results were negative. Videourodynamics did not show urodyanamically proven SUI or detrusor overactivity.

## Discussion

To the extent of our knowledge, this report is the first to mention an unusual cystoscopic finding of a “Central Road,” a cord‐like appearance in the center of the bladder trigon and posterior wall, in a woman with deteriorating incontinence following LSC. Although a cystoscopy is not regarded as a mandatory examination to evaluate urinary incontinence, it is helpful to exclude tumors, stones, and especially mesh exposure in patients with a past history of urogynecological surgery using mesh. In this case, we found no mesh exposure but a “Central Road” cystoscopic finding, which can be an indication of mesh overtensioning.

Sacrocolpopexy, suspension of the vaginal walls to the sacrum’s anterior longitudinal ligament using synthetic mesh, is a durable surgical alternative to treat POP.^3^ In the past, open surgery (abdominal sacrocolpopexy) was regarded as a gold standard. Now, LSC including robot‐assisted LSC is becoming more popular due to its low invasiveness. After the 2011 FDA alert regarding mesh complications following TVM, a significantly greater number of surgeons have begun to use LSC.^2^ Surgeons should be aware of LSC complications as they occur more often in the beginning of the learning curve.

All kinds of prolapse surgeries are known to worsen or cause de novo SUI by eliminating obstructions, but there are also iatrogenic factors including surgical techniques.^4^ It has been a topic of discussion among professionals that excessive tension in a prolapse repair leads to the opening of the bladder neck and worsened urinary incontinence. LeClaire *et al*. reported that a larger decrease in point Aa was de novo SUI’s risk factor.^5^ Miwa *et al*. reported that the retrovaginal angle measured by transperitoneal ultrasound was significantly enlarged postoperatively in patients with worsened SUI.^6^

In our case, a “Central Road” cystoscopic finding and cystourethrography indicated that the LSC mesh pulled the posterior vesical wall excessively in the direction of the sacrum. Many surgeons prefer to dissect the vaginal walls as distally as possible and fix them with mesh to achieve full suspension of the vaginal walls. However, overtensioning of such LSC mesh toward the sacrum can cause excessive straightening (dekinking) of the bladder neck and proximal urethra, thus worsening SUI and possibly UUI (stress‐induced instability). There are three ways to restore continence in such cases: (i) midurethral sling procedures, (ii) laparoscopic loosening of the LSC mesh, and (iii) both. A favorable Bonny test in our case suggested that a midurethral sling would correct excessive straightening of the bladder neck and urethra caused by the LSC mesh. As a midurethral sling is a less invasive procedure than loosening LSC mesh, a midurethral sling could proceed laparoscopic loosening.

## Conclusions

Overtensioning of the LSC mesh resulting in a “Central Road” cystoscopic finding can lead to dekinking of the bladder neck and the urethra and intensify urinary incontinence. Thus, it is imperative that we pay attention to the mesh tension adjustment during LSC.

## Conflict of interest

The authors declare no conflict of interest.

## Author contributions

Kumiko Kato: conception and design, acquisition of data, analysis and interpretation of data, and drafting of the manuscript. Yasukuni Yoshimura and Masahiro Narushima: sonception and design and analysis and interpretation of data. Shoji Suzuki: acquisition of data and analysis and interpretation of data. Ryohei Hattori: supervision.

## References

[1] Murphy M , Holzberg A , van Raalte H *et al* Pelvic Surgeons Network. Time to rethink: an evidence‐based response from pelvic surgeons to the FDA safety communication: “UPDATE on serious complications associated with transvaginal placement of surgical mesh for pelvic organ prolapse.” Int. Urogynecol. J. 2012; 23: 5–9. 22086260 10.1007/s00192-011-1581-2

[2] Obinata D , Sugihara T , Yasunaga H *et al* Tension‐free vaginal mesh surgery versus laparoscopic sacrocolpopexy for pelvic organ prolapse: Analysis of perioperative outcomes using a Japanese national inpatient database. Int. J. Urol. 2018; 25: 655–9. 29729035 10.1111/iju.13587

[3] Nygaard I , Brubaker L , Zyczynski HM *et al* Long‐term outcomes following abdominal sacrocolpopexy for pelvic organ prolapse. JAMA 2013; 309: 2016–24. 23677313 10.1001/jama.2013.4919 PMC3747840

[4] Fatton B . Is there any evidence to advocate SUI prevention in continent women undergoing prolapse repair? An overview. Int. Urogynecol. J. 2009; 20: 235–45. 10.1007/s00192-008-0734-4 18936868

[5] LeClaire EL , Mukati MS , Juarez D *et al* Is de novo stress incontinence after sacrocolpopexy related to anatomical changes and surgical approach? Int. Urogynecol. J. 2014; 25: 1201–6. 24647867 10.1007/s00192-014-2366-1

[6] Miwa K , Moriyama Y , Ito H *et al* The relationship between de novo stress urinary incontinence and mesh fixation technique on laparoscopic sacrocolpopexy. J. Jap. Continence Soc. 2017; 28: 351–5.

